# Oxidation Mechanism of Core-Shell Structured Al@PVDF Powders Synthesized by Solvent/Non-Solvent Method

**DOI:** 10.3390/ma15093036

**Published:** 2022-04-22

**Authors:** Chuanbin Wang, Mei Qin, Zhuoran Yi, Haoyuan Deng, Junjie Wang, Yi Sun, Guoqiang Luo, Qiang Shen

**Affiliations:** 1 State Key Lab of Advanced Technology for Materials Synthesis and Processing, Wuhan University of Technology, Wuhan 430062, China; wangcb@whut.edu.cn (C.W.); 290912@whut.edu.cn (M.Q.); fgmyzr123@whut.edu.cn (Z.Y.); 290787@whut.edu.cn (H.D.); 290687@whut.edu.cn (J.W.); luogq@whut.edu.cn (G.L.); sqqf@whut.edu.cn (Q.S.); 2 Chaozhou Branch of Chemistry and Chemical Engineering Guangdong Laboratory, Chaozhou 521000, China

**Keywords:** aluminum, polyvinylidene fluoride, solvent/non-solvent method, core-shell structured, energy release

## Abstract

Micron-sized aluminum (Al) powders are extensively added to energy-containing materials to enhance the overall reactivity of the materials. However, low oxidation efficiency and energy release limit the practical application of Al powders. Polyvinylidene fluoride (PVDF), the most common fluoropolymer, can easily react with Al to form aluminum fluoride (AlF_3_), thus promoting the oxidation of Al powders. In this paper, core-shell structured Al@PVDF powders were synthesized by solvent/non-solvent method. Thermal analysis results show that the weight and exothermic enthalpy of Al@PVDF powders are 166.10% and 11,976 J/g, which are superior to pure Al powders (140.06%, 6560 J/g). A detailed description of the oxidation mechanisms involved is provided. Furthermore, constant volume pressure results indicate that Al@PVDF powders have outstanding pressure output ability in the environment of 3 MPa oxygen. The study provides a valuable reference for the application of Al powders in energetic materials.

## 1. Introduction

As the most abundant metallic element on earth, Al is widely applied in fireworks, explosives, and solid propellants owing to its low price and high energy density (31 kJ/g) [1,2,3,4]. However, the large specific surface area and high reactivity of Al powders make them prone to reacting with oxygen and water in the air to form a dense oxide layer during production and storage [5,6,7], which hinders the diffusion of oxygen and reduces combustion and oxidation efficiency [8,9].

To improve the oxidation properties and stability of Al powders at room temperature, scientists have tried various materials as surface coating agents, such as carbon [10], hydroxyl-terminated polybutadiene (HTPB) [11], nitrocellulose (NC) [12], perfluoropolyether (PTFE) [13], polyvinylidene fluoride (PVDF) [14], Ni [15], Co [16], Viton, and THV [17]. Among these materials, fluorocarbons have attracted intense interest owing to their excellent mechanical properties and thermal stability. Recent studies have demonstrated that the reaction of fluorinated groups with oxides generates AlF_3_ with a large amount of energy and that the generation of AlF_3_ provides an efficient pathway for the diffusion of oxygen [18,19,20,21].

Polyvinylidene fluoride (PVDF), containing 59 wt% fluorine, is widely employed for its excellent chemical stability and corrosion resistance to acid and alkali. PVDF is soluble in several solvents, such as *N*,*N*-dimethylformamide (DMF), and dimethyl sulfoxide (DMSO) [22]. Various Al/PVDF matrix materials were fabricated by diverse methods. Lyu et al. [23] prepared PVDF/CuO/Al nanocomposites by the electrospinning technique. The obtained materials showed improved heat release, oxidation capability, and reaction efficiency. Wang et al. [24] produced Al/PVDF films by 3D printing, electrospray (E-spray), and electrospin (E-spin). The study revealed that optimization of Al/PVDF materials structure is essential for energy release. Chen et al. [25] prepared the Al/PVDF films by phase inversion with high combustion performance. Ji et al. [26] obtained core-shell structured Al@PVDF particles by applying the evaporation method. Results showed that Al@PVDF particles have outstanding pressure output ability and excellent combustion performance compared to pure Al particles. Ke et al. [27] used vacuum freeze-drying technology to produce Al/PVDF nanoenergetic films, and a synergistic effect was found between the PVDF and aluminum nanoparticles (Al NPs) during the reaction process. Kim et al. [14] synthesized PVDF/Al particles by using the spontaneous induced fluorination reaction on the surface of Al particles as a medium and made a suggested mechanism for the oxidation reaction of PVDF/Al particles, which is important for the application of micro-Al powders.

However, Al/PVDF matrix energetic materials with a core-shell structure have not yet been synthesized by solvent/non-solvent method. The solvent/non-solvent method [28] is where the difference in solubility of a solute in different solvents enables the substance to precipitate on the surface of another substance in the crystallized or encapsulated form. This method is extensively utilized in the research and application of energetic materials, especially for the refinement and preparation of materials for core-shell structure. In our present work, soluble PVDF was coated on the surface of spherical Al particles via solvent/non-solvent method to investigate its effect on the thermal oxidation of Al powders. The morphology and structure of the PVDF-coated Al powders were characterized by field-emission scanning electron microscopy (FE-SEM) and field-emission transmission electron microscopy (FE-TEM). Results of thermal properties were collected by thermogravimetry and differential scanning calorimeter (TG-DSC). The phases and crystal structure of the samples and their oxidation products were analyzed with X-ray diffraction (XRD). It was found that the PVDF layer significantly enhances the oxidation efficiency and heat release of the Al powders. The research provides a feasible approach for promoting the development of the micro-Al powders applied in energetic materials.

## 2. Experimental Procedures

### 2.1. Materials

Al powders (average size of 5.0 μm) were obtained from Yuanyang Powder Technology Co., Ltd, Xinxiang, China. Polyvinylidene fluoride (PVDF) powders (Mw ≈ 476,000) were purchased from Micxy Chemical Co., Ltd, Chengdu, China. *N*,*N*-dimethylformamide (DMF, 99.8%) and anhydrous ethanol (99.8%) were bought from Sinopharm Chemical Reagent Co., Ltd., Shanghai, China.

### 2.2. Preparation of Al@PVDF Powders

In this experiment, 80 mg PVDF powders (8% weight of Al powders) were dissolved in 20 mL DMF solution and magnetically stirred for 10 min to obtain a completely dissolved PVDF solution. The mixed solution was acquired with 20 mL ultrapure water and 20 mL anhydrous ethanol solution, and 1 g Al powders was dispersed in it. Then, the mixed dispersion was pretreated through an ultrasonic homogenizer to prepare a stable Al suspension. PVDF solution was added dropwise to the Al suspension with a rate of 2 mL/min. As non-solvent for the PVDF, the water and alcohol caused the PVDF particles to precipitate out of the DMF solution and adsorb onto the surface of Al particles, forming a protective layer. PVDF-coated Al powders were washed three times with an ethanol solution, filtered, and dried under vacuum at 60 °C. The collected sample was named Al@PVDF powders.The Al/PVDF powders prepared from Al suspension without pretreatment by ultrasonic homogenizer are employed for comparison with Al@PVDF powders prepared from pretreated Al suspension.

### 2.3. Characterization

The morphology and structure of the Al@PVDF powders were characterized by field-emission scanning electron microscope (FE-SEM, TESCAN, MIRA3, Brno, Czech) and field-emission transmission electron microscopy (FE-TEM, JEOL, JEM-2100F, Tokyo, Japan). Thermogravimetric (TG) and differential scanning calorimetry (DSC) experiments were performed on simultaneous thermal analyzer (NETZSCH, STA449F5, Bavaria, Germany) under 50 mL/min of air flow (N_2_/O_2_ 79/21 vol.%) and 20 mL/min of Ar flow (protective gas). Next, 5–10 mg samples were placed into an alumina pan and heated at a rate of 10 °C/min. Functional group information of samples was carried out through Fourier transformed infrared spectroscopy (FTIR, Thermo Fisher Scientific Co., Nicolet 6700, Waltham, MA, USA). The wavenumber range of infrared spectra is 4000–400 cm^−1^. The phases and crystal structure of samples and their oxidation products recovered from TG-DSC analysis were characterized by X-ray diffraction (XRD, Panalytical, Empyrean, Eindhoven, The Netherland). To investigate the pressure output capability of the Al@PVDF powders, the pressure chamber test (PCT, Hbst. Co., Ltd, HB-CVC01, Mianyang, China) was performed. The samples (200 mg) were placed in a closed container and ignited by flame ignition method in an oxygen environment at 3 MPa.

## 3. Results and Discussions

### 3.1. Morphology and Composition of Al@PVDF Powders

**Figure 1:**
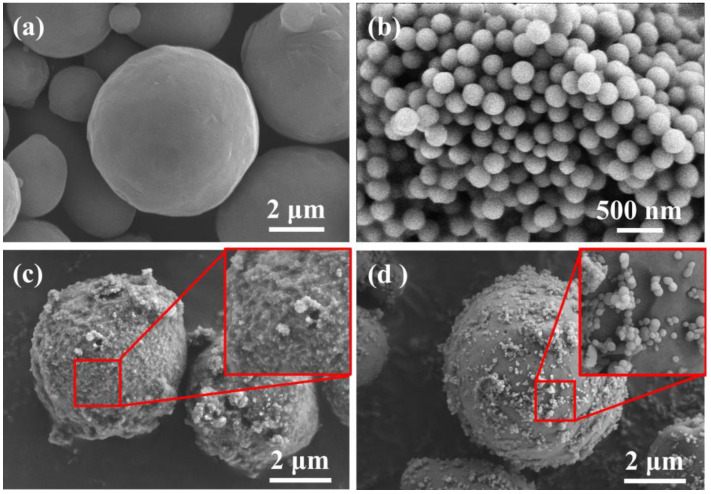
SEM images of Al powders (**a**), PVDF powders (**b**), Al@PVDF powders (**c**), and Al/PVDF powders (**d**).

**Figure 2:**
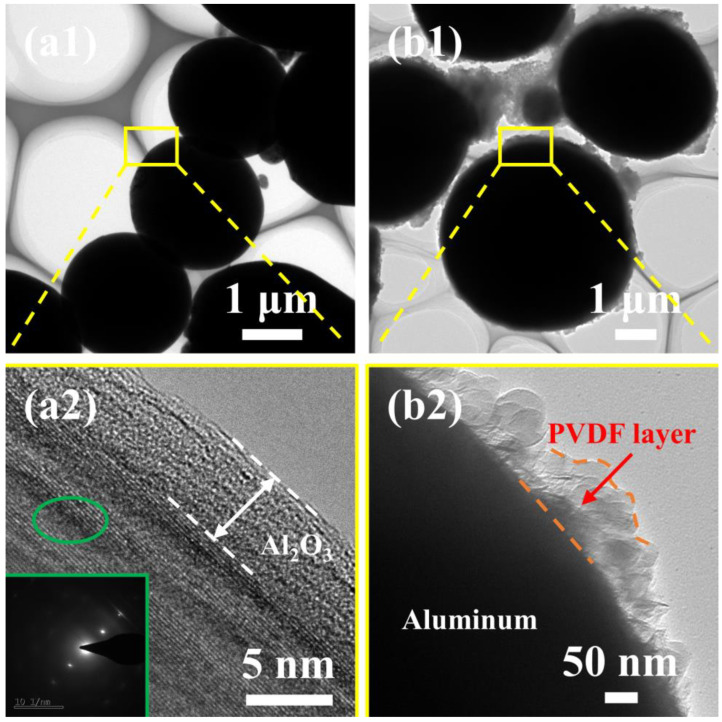
TEM images of Al powders (**a1**) and Al@PVDF powders (**b1**), enlarged TEM image of the Al_2_O_3_ region (**a2**), enlarged TEM image of PVDF-coated region (**b2**).

Figure 1 displays SEM images of the raw materials and PVDF-coated Al powders. It can be observed that the surface of spherical Al powders was smooth, and the particles were well dispersed. The PVDF particles were bonded to each other, and the particle size was approximately 200 nm. As shown in Figure 1c, for Al@PVDF powders, a variety of PVDF particles precipitation from non-solvents were adsorbed onto the surface of Al particles, and uniformly dispersed to form a complete coating, while for Al/PVDF powder, Figure 1d shows that PVDF particles were scattered on the surface of Al particles. TEM images of Al@PVDF powders and Al powders are shown in Figure 2. The thickness of amorphous Al_2_O_3_ is approximately 3–5 nm. Al@PVDF particles exhibit a typical core-shell structure, as shown in Figure 2b2. Moreover, a uniform PVDF layer could promote the reaction of Al with PVDF.

**Figure 3:**
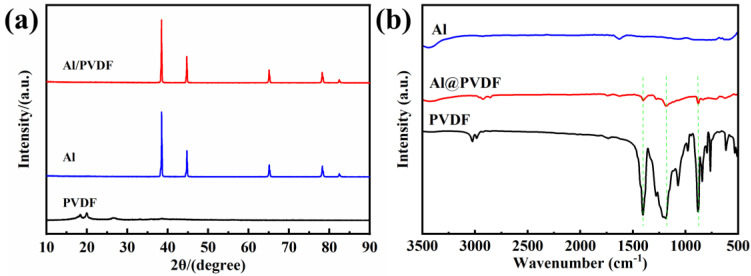
XRD patterns (**a**) and FTIR spectra (**b**) of Al powders, Al@PVDF powders, and PVDF powders.

To further investigate the composition and structure of Al@PVDF powders, Figure 3 displays the XRD and FTIR results. In the XRD pattern of Al@PVDF powders, the intense diffraction peaks at 38.5°, 44.7°, 65.1°, 78.2°, and 82.5° can be recognized as the (111), (200), (220), (311), and (222) crystal planes of metallic Al, respectively. Furthermore, the characteristic peaks at 18.3°, 19.9°, and 26.7° derived from the α-phase of pure PVDF powders are hardly detected in Al@PVDF powders. We speculate that the PVDF content in Al@PVDF powders is low. In Figure 3b, three characteristic peaks at 1404 cm^−1^, 1188 cm^−1^, and 880 cm^−1^ are recorded. Three peaks were derived from the in-plane bending vibration of the -CH_2_ group, the stretching vibration of the CF_2_ group, and the skeleton vibration of the C-C bond, respectively. The results provide evidence for the adhesion of PVDF particles onto the surface of Al particles. There is no doubt that PVDF was successfully coated on the Al surface by solvent/non-solvent method.

### 3.2. Thermal Properties of Al@PVDF Powders

**Figure 4:**
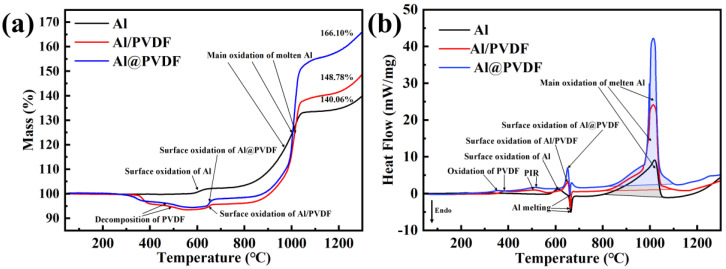
TG and DSC curves of Al powders, Al/PVDF powders, and Al@PVDF powders, (**a**) TG, (**b**) DSC.

Figure 4 shows the TG and DSC curves of Al powders, Al/PVDF powders, and Al@PVDF powders under air atmosphere from 40 to 1300 °C at a heating rate of 10 °C/min. Both PVDF-coated Al powders exhibited superior thermal properties compared to Al powders. As is shown in Figure 4a, there was no significant change in the initial weight gain of Al powders and PVDF-coated Al powders at 100 °C, suggesting that the PVDF-coated Al powders are thermally stable at room temperature. The oxidation process of micron-Al powders at 40–1300 °C could be separated into four stages, as shown in the TG curve. The first stage ranges from 40 to 550 ℃, where the thickness of the amorphous oxide layer increases and oxidation is extremely weak. The second stage is at 550–670 °C, during which the amorphous oxide transforms to γ-Al_2_O_3_. The density of γ-Al_2_O_3_ (3.65 g/cm^3^) is higher than that of the amorphous state (3.1 g/cm^3^). Thus, the newly formed γ-Al_2_O_3_ cannot form a continuous and complete shell on the surface of Al, and a great number of cracks emerge in the oxide shell. These cracks and the grain boundaries generated in the form of a fast diffusion network in the crust structure facilitate the diffusion of O_2,_ and quick oxidation of the Al corresponds to an increase of weight in the TG curve. Through continuous oxidation, the rate of oxidation reduces when the cracks in the oxide layer are repaired, and the γ-Al_2_O_3_ layer covers the surface of Al. The third stage is from 670 to 1050 °C. An intense oxidation reaction called the main reaction happens in this period. During the main reaction stage, the expansion of the volume exerts tensile stress on the oxide shell caused by the difference in density between the molten Al (2400 kg/m^3^) and the solid Al (2700 kg/m^3^), while the transformation of γ-Al_2_O_3_ to α-Al_2_O_3_ induces the shrinkage of the oxide shell surface [29]. It is the shrinkage of the oxide shell and the tension of the volume expansion that leads to the fracture of the alumina shell. The molten Al penetrates through the voids and cracks to contact the oxygen and undergoes a rapid oxidation reaction. The vigorous oxidation reaction is accompanied by a great amount of energy release and a sudden increase in weight gain. When the formed α-Al_2_O_3_ covers the surface again, the internal Al is protected. The fourth stage of the oxidation process occurs beyond 1100 °C, where active Al inside continues to react at a reduced rate [30]. When the temperature raised to 1300 °C, the weight of Al powders increased by 140.06%. The weight of the Al@PVDF powders and Al/PVDF powders decreases before 550 °C due to the decomposition of the PVDF. The oxidation process for PVDF-coated powders in the second and fourth periods is identical to that of Al powders. However, the oxidation of PVDF-coated Al powders is enhanced by the addition of PVDF during the main reaction stage, especially for Al@PVDF powders. At 1300 °C, the weight of Al/PVDF powders and Al@PVDF powders is increased by 148.78% and 166.10%, respectively.

In Figure 4b, two small exothermic peaks occur in the temperature range of 350–450 °C and 450–550 °C on the DSC curves of Al@PVDF powders and Al/PVDF powders, respectively, which are ascribed to the decomposition of PVDF and the fluorination of Al. The DSC curves of all samples show an obvious exothermic peak at 550–650 °C, which results from surface oxidation due to the transformation of the crystalline form of Al_2_O_3_. An exothermic peak situated around 660 °C corresponds to the melting of Al. Nevertheless, for PVDF-coated Al powders, the heat absorbed by melting is considered to be slightly balanced with the heat released by oxidation. The distinct broad peak observed on the DSC curve of Al powders at 800–1050 °C is consistent with the weight increase on the TG curve. Overflowing molten Al reacted with O_2_ to form Al_2_O_3_ covering the surface and releasing substantial energy. The exothermic enthalpy of the main reaction of the Al powders is calculated by the analysis software to be only 6560 J/g. In the third stage, the energy release of the PVDF-modified Al powder is greatly enhanced by the combined effect of the sublimation of the AlF_3_, the shrinkage of the oxide shell, and the volume expansion caused by the melting of Al [31]. The sharp peaks of the PVDF-coated Al powders reveal a faster oxidation reaction than Al powders with a broad peak. The exothermic enthalpies of the main exothermic peaks near 1000 °C are calculated to be 11,976 J/g and 9452 J/g for Al@PVDF powders and Al/PVDF powder, respectively. The results of TG-DSC imply that the addition of PVDF could remarkably facilitate the oxidation activity and increase the exothermic enthalpy of Al powders. In contrast, Al@PVDF powders with a core-shell structure exhibit more striking mass gain and energy release. This may be because the homogeneous and well-defined interfaces between the reactants are essential in improving performance [23,32]. As for Al@PVDF particles with the core-shell structure, shortened diffusion distance and larger contact area result in improved thermal performance. The results of TG-DSC imply that the addition of PVDF could remarkably facilitate the oxidation activity and increase the exothermic enthalpy of Al. In particular, Al@PVDF powders with a core-shell structure exhibit preferable thermal properties.

### 3.3. Oxidation Mechanism of Al@PVDF Powders

**Figure 5:**
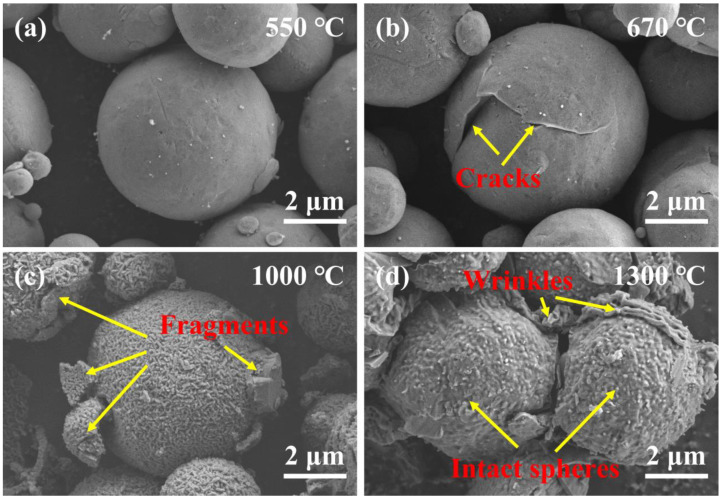
SEM images of oxidation products of Al powders at different temperatures. (**a**) 550 °C; (**b**) 670 °C; (**c**) 1000 °C; (**d**) 1300 °C. The products were recovered from TG-DSC analysis.

**Figure 6:**
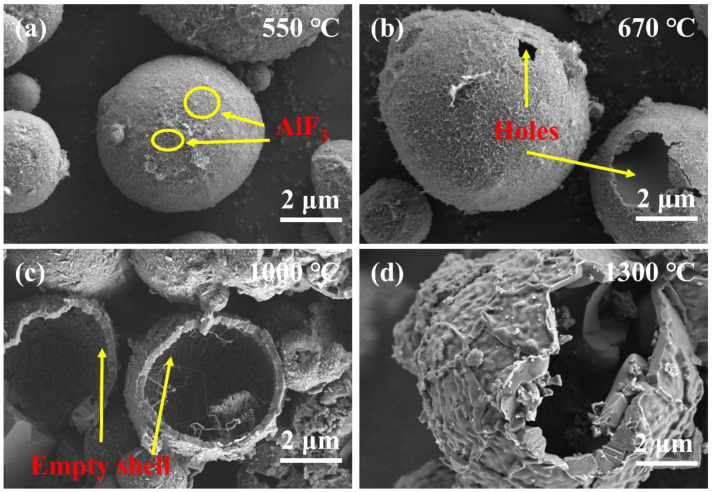
SEM images of oxidation products of Al@PVDF powders at different temperatures. (**a**) 550 °C; (**b**) 670 °C; (**c**) 1000 °C; (**d**) 1300 °C. The products were recovered from TG-DSC analysis.

To explore the oxidation process of Al@PVDF powders, the oxidation products of the samples were recovered and analyzed at different temperatures. Figure 5 depicts the SEM images of oxidation products. At about 550 °C, the surface of Al enjoys high smoothness. The PVDF decomposed before 550 °C and reacted with Al to form AlF_3_, which attached to the Al surface. At 550–670 °C, micro-cracks and fissures appear on the surface of t Al spheres as the result of the transformation of amorphous alumina into γ-Al_2_O_3_ [33]. Cracks are observed in Figure 5b. At approximately 1000 °C, driven by shrinkage of the oxide shell and the tension of the volume expansion, the molten Al breaks through the oxide layer and penetrates through the voids and cracks to contact with O_2_. Dense α-Al_2_O_3_ is formed and covers the surface of Al [29]. As is shown in Figure 6, there are some fragments of oxide on the surface of Al@PVDF particles, and a portion of the particles turned into empty Al_2_O_3_ shells. A plausible explanation is that the sublimation of AlF_3_ and the stress generated by the molten Al droplets motivate Al cores to break the confines of the oxide layer. That means a shell-breaking reaction occurs at 1000 ℃. Subsequently, the internal Al droplets erupt rapidly and come into contact with oxygen. For Al@PVDF powders, the sublimation of AlF_3_ and the oxidation of Al are mutually reinforcing during the vigorous oxidation reaction. The sublimation of AlF_3_ promotes the intense oxidation of Al, while the heat released by the strong oxidation makes the AlF_3_ disappear at a temperature below the sublimation point (1277 °C) [31]. As the temperature rises to 1300 ℃, volume contraction associated with the transformation of the different crystalline forms of Al_2_O_3_ contributes to the slow oxidation of Al. Similarly, for Al@PVDF powders, the slow oxidation of internal Al increases the thickness of the oxide shell.

**Figure 7:**
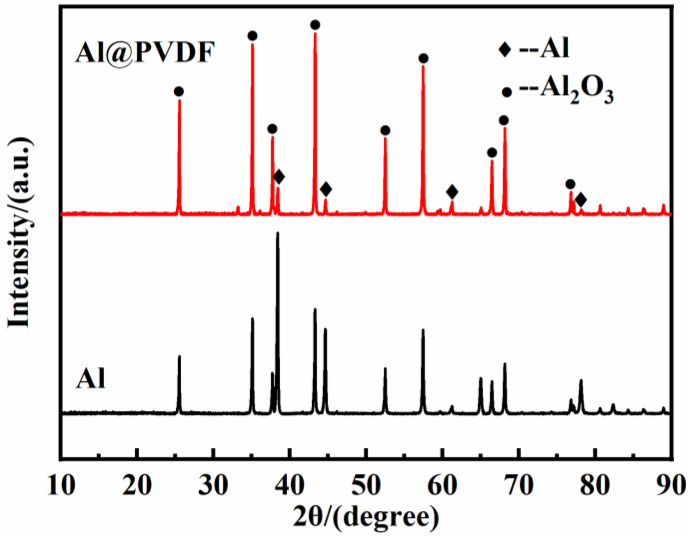
XRD patterns of oxidation products at 1300 ℃. The products were recovered from TG-DSC analysis.

XRD was employed to identify the crystallographic structure of oxidation products of Al@PVDF powders and Al powders after being heated to 1300 °C. The products were recovered from TG-DSC analysis. Figure 7 reveals diffraction peaks of Al_2_O_3_ and Al are detected in the Al@PVDF powders the Al powders, implying that both samples underwent an inadequate oxidation reaction with a small amount of Al remaining up to 1300 °C. In contrast, the peak intensity of Al for the oxidation products of Al@PVDF powders is lower than that of Al powders. The XRD results are consistent with the trend of the TG curves.

**Figure 8:**
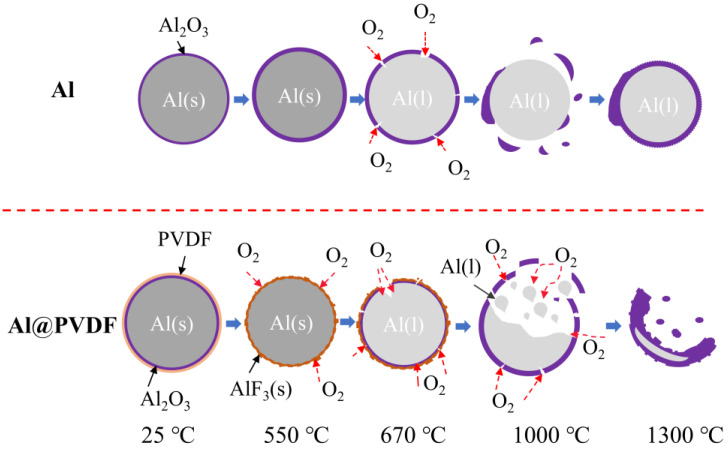
The diagram of the potential oxidation mechanism.

Based on the SEM, TG-DSC, and XRD results, a potential oxidation mechanism is proposed, as shown in Figure 8. For Al powders, the main oxidation relies on destroyed crust motivated by the excitation of stresses generated by the shrinkage of the oxide shell and the expansion of the Al core. The newly generated oxide layer quickly covers the surface of molten Al droplets, preventing further oxidation. Consequently, plenty of active Al remains. For Al@PVDF powders, the reasons for oxidation below 670 °C are the same. However, at around 1000 °C, there is sublimation of AlF_3_, tension arising from shrinkage of the crust, and expansion of the melting tear the oxide shell. The spattered Al droplets react immediately upon contact with oxygen. Meanwhile, the active Al retained in the oxide shell is further oxidized, resulting in an increase in the thickness of the oxide layer. Consequently, the oxidation efficiency and energy release of the Al@PVDF powders are significantly improved.

### 3.4. Pressure Output Capacity of Al@PVDF Powders

**Figure 9:**
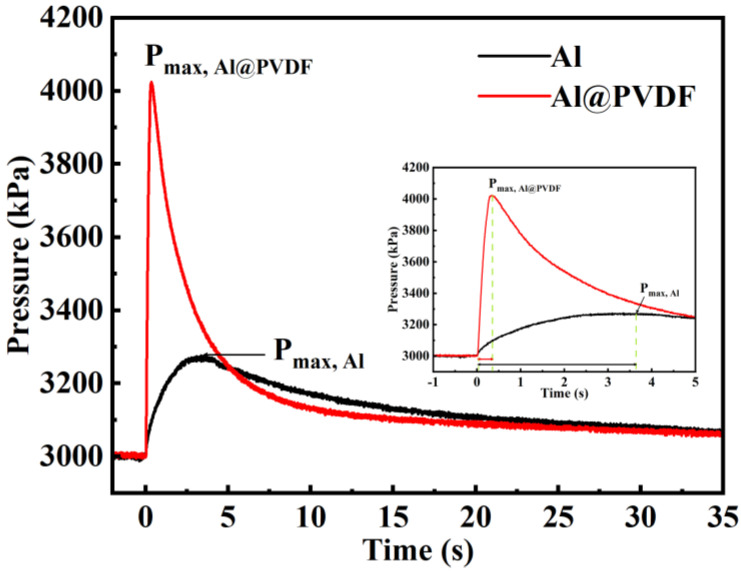
Comparison of the pressure change as a function of time. Inset: enlarged P-T curves in the time range of 0–5 s.

Figure 9 presents the pressure variation over time documented by collecting pressure data in a closed chamber. The maximum pressure (P_max_) of Al@PVDF powders (4025 kPa) is higher than that of Al powders (3274 kPa), which is due to the decomposition of PVDF produces gaseous phases such as CO_2_ and HF. The inset shows the enlarged P-T curves in the time range of 0–5 s. It can be observed that the ignited pressure of Al@PVDF powder achieves P_max_ at 0.37 s, while Al powders take nearly 10 times longer to reach it.

## 4. Conclusions

In this paper, we succeeded in preparing PVDF-coated Al powders with a core-shell structure by solvent/non-solvent method. The PVDF layer not only acts as a protective shell to protect the internal active Al from oxidation and corrosion at room temperature but also as an oxidation activator. The obtained Al@PVDF powders exhibit higher weight gain and energy release than Al powders. The mass of Al@PVDF powders and Al powders after being heated to 1300 °C are 166.10% and 140.06%, respectively, and the exothermic enthalpy in the main reaction stage are 11,976 J/g and 6560 J/g, respectively. Furthermore, a potential oxidation mechanism of Al@PVDF powders was proposed. The result of constant volume pressure reveals that Al@PVDF powders have excellent pressure output capacity. It can be expected that Al@PVDF powders with a core-shell structure prepared by solvent/non-solvent method would be widely employed in the field of energetic materials.
